# El Niño-Southern Oscillation Is Linked to Decreased Energetic Condition in Long-Distance Migrants

**DOI:** 10.1371/journal.pone.0095383

**Published:** 2014-05-02

**Authors:** Kristina L. Paxton, Emily B. Cohen, Eben H. Paxton, Zoltán Németh, Frank R. Moore

**Affiliations:** 1 Department of Biological Sciences, The University of Southern Mississippi, Hattiesburg, Mississippi, United States of America; 2 Smithsonian Conservation Biology Institute, Migratory Bird Center, National Zoological Park, Washington, DC, United States of America; 3 Pacific Island Ecosystems Research Center, United States Geological Survey, Hawaii National Park, Hawaii, United States of America; 4 Department of Neurobiology, Physiology, and Behavior, University of California Davis, Davis, California, United States of America; Utrecht University, Netherlands

## Abstract

Predicting how migratory animals respond to changing climatic conditions requires knowledge of how climatic events affect each phase of the annual cycle and how those effects carry-over to subsequent phases. We utilized a 17-year migration dataset to examine how El Niño-Southern Oscillation climatic events in geographically different regions of the Western hemisphere carry-over to impact the stopover biology of several intercontinental migratory bird species. We found that migratory birds that over-wintered in South America experienced significantly drier environments during El Niño years, as reflected by reduced Normalized Difference Vegetation Index (NDVI) values, and arrived at stopover sites in reduced energetic condition during spring migration. During El Niño years migrants were also more likely to stopover immediately along the northern Gulf coast of the southeastern U.S. after crossing the Gulf of Mexico in small suboptimal forest patches where food resources are lower and migrant density often greater than larger more contiguous forests further inland. In contrast, NDVI values did not differ between El Niño and La Niña years in Caribbean-Central America, and we found no difference in energetic condition or use of coastal habitats for migrants en route from Caribbean-Central America wintering areas. Birds over-wintering in both regions had consistent median arrival dates along the northern Gulf coast, suggesting that there is a strong drive for birds to maintain their time program regardless of their overall condition. We provide strong evidence that not only is the stopover biology of migratory landbirds influenced by events during the previous phase of their life-cycle, but where migratory birds over-winter determines how vulnerable they are to global climatic cycles. Increased frequency and intensity of ENSO events over the coming decades, as predicted by climatic models, may disproportionately influence long-distance migrants over-wintering in South America.

## Introduction

Global climatic patterns and their influence on local weather parameters such as precipitation and temperature greatly impact biological systems, including the composition and distribution of habitats, as well as determine the abundance of resources available to higher trophic levels [1], [2]. Long-distance migratory animals may be particularly vulnerable to fluctuations in global climate patterns as their seasonal movements between multiple geographic regions increase the likelihood that they will encounter unfavorable or extreme weather patterns at some point during their annual cycle. Further, climatic events that affect an individual during one phase of the annual cycle often carry-over to influence an individual's success in following seasons, resulting in climatic conditions in one part of the world affecting population dynamics in multiple dispersed regions [3], [4]. Our ability to predict how migratory animals will respond to changing climatic conditions requires integrating how climatic conditions during different phases of the annual cycle and at different geographic locations interact to influence overall population dynamics [5]–[7]. That said, establishing how events at any one stage of the annual cycle influence an individual's performance in subsequent seasons is one of the greatest research challenges of our time [8]. 

El Niño Southern Oscillation (ENSO) is a major climatic phenomenon influencing weather patterns around the world, and in terrestrial ecosystems exerts both direct and indirect impacts on plant productivity and community dynamics through its effects on precipitation. Periodic oscillations between El Niño (warm state) and La Niña (cold state) episodes results in dramatic increases in rainfall in some regions, while creating severe droughts in other areas [1]. Research examining the impacts of ENSO events on migratory landbird populations has primarily been limited to the stationary periods (e.g., breeding and over-wintering; [3], [9]–[11]), while largely overlooking the role that the migratory period contributes to the overall annual cycle (but see [12], [13]). Yet, for migratory species the negative impacts of climatic events during the stationary periods may not be manifested until migration, amplifying the risk of mortality during migration, the phase of the annual cycle most often thought to be limiting many migratory species [14]–[16]. Climatic models predict the frequency and intensity of ENSO events will increase over the coming decades [17], [18], [19], which could dramatically alter habitat conditions at breeding, over-winter, and migratory stopover sites from year to year with cascading impacts on the migratory time period, underscoring the critical need for knowledge about the linkages between climatic events during the stationary phases and factors that influence the success of migration.

During migration birds are faced with numerous challenges, including increased energy demands, acquisition of food in unfamiliar habitats, predator avoidance, intra- and inter-specific competition for limited resources, abiotic perturbations, and navigational challenges [20]. These challenges are intensified by the time constraints associated with migration and the negative fitness consequences that can occur with delayed arrival on the breeding grounds [21]. The residual effects of events in the previous season can influence how well a migrant responds to challenges encountered during migration, measured in terms of time and condition [22]. For example, drier and hotter climatic conditions during the over-winter period may alter the availability of food resources during a time period critical for the preparation of spring migration [23], [24]. A migrant departing the nonbreeding grounds with insufficient energy stores does not have a buffer against adverse weather encountered en route or scarcity of food during stopover, either or both of which could affect the migrant's time schedule [25], [26] and exposure to sources of mortality. Therefore, carry-over effects of climatic events experienced during the stationary period may affect spring migration success, with cascading impacts on reproductive success during the subsequent breeding season.

We utilized a unique long-term migration dataset collected on the northern coast of the Gulf of Mexico (GOM) to understand how ENSO climatic events that differentially impact two tropical over-winter regions carry-over to affect intercontinental bird migrants during spring passage. The movement of birds across the GOM is a conspicuous feature of the Nearctic- Neotropical bird migration system, and tens of millions of birds stopover in coastal habitats each spring to rest and refuel [20]. This study provides a broad-scale examination of how ENSO events in geographically distinct regions of the Western Hemisphere carry-over to impact the migratory success of several intercontinental migratory bird species, filling an important gap in our understanding of how global climatic cycles influence migratory species throughout their annual cycle.

We predicted that El Niño events will differentially impact migratory birds over-wintering in South America and Caribbean-Central America because of different precipitation and temperature patterns in these regions. During the winter months in northern South America El Niño events consistently cause hotter and drier environmental conditions, while La Niña events result in cooler and wetter environmental conditions [27]. In contrast, El Niño events overall for the Caribbean-Central America region results in only a weak tendency towards drier environmental conditions, especially compared to neighboring regions such as South America [27]. However, localized areas within the Caribbean-Central America region are affected by El Niño events, particularly regions within the Caribbean and the Pacific coast of Central America [28], [29]. To directly link ENSO climatic patterns with environmental conditions experienced by migratory birds at over-wintering sites in South America and Caribbean-Central America, we measured changes in vegetation greenness, via the Normalized Difference Vegetation Index (NDVI), between El Niño, La Niña, and non-ENSO events for species-specific over-winter ranges. We then quantified the effects of ENSO events on factors important to migratory success such as a bird's migratory condition, timing of migration, and the number of birds utilizing coastal habitat. Specifically, we predicted limited influence of ENSO events on migratory condition for birds over-wintering in the Caribbean and Central America given the overall weak impact of ENSO events in these regions. In contrast, we predicted that the drier, warmer El Niño conditions experienced by migratory birds over-wintering in South America will result in: a) Migratory birds having lower energetic conditions during spring migration. b) Lower energetic conditions will result in more birds utilizing coastal habitats immediately after their non-stop flight across the GOM where food resources are lower and migrant density greater than in hardwood forests further inland [30], [31]. c) Bird will be less likely to maintain their time program given reduced energetic conditions, resulting in an overall delay in spring migration.

## Materials and Methods

### Ethics statement

The research conducted for this study was carried out in accordance with the Ornithological Council's guidelines for the use of wild birds in research and was approved by The University of Southern Mississippi's institutional animal care and use committee (protocol #1092210). Other permits were from the United States Department of the Interior bird banding laboratory (permit #21221) and Fish and Wildlife Service (permit #MB75836-3) and the Louisiana Department of Wildlife and Fisheries (permit #LNHP-11-058).

### Study Area and Species Selection

We evaluated the potential carry-over effects of ENSO events experienced at over-winter sites on spring migrants at a long-term migration station located along the northern coast of the GOM in Cameron Parish, Louisiana (29°45′N, 93°37′W). Coastal woodlands represent critical stopover habitat for Nearctic-Neotropical landbird migrants [30], [32], providing the first landfall for eastern migrants in the spring after non-stop flight across this ecological barrier. Birds were captured with mist-nets (12×2.6 m, 30 mm mesh) on a daily basis, 28 March to 6 May, for approximately 8 hrs (0800 to 1700) during all years included in this study (1993 to 1996, 1998 to 2010). Upon capture, birds were banded with a USGS band, age and sex determined according to Pyle [33], subcutaneous fat assessed according to Helms and Drury [34], and weighed to nearest 0.1 g with an electronic scale. We chose species that over-winter in either Caribbean-Central America region or South America to test the hypothesis of differential effects of ENSO events in these regions. Within each region, focal species were selected that did not breed at the stopover site and had 30 or more captures per year for at least 10 years of the study (some species captures fall below 30 captures when the overall data set was restricted for some analyses; see Table 1). Focal species over-wintering in Caribbean-Central America were: Wood Thrush (WOTH; *Hylocichla mustelina*), Ovenbird (OVEN; *Seiurus aurocapillus*), Hooded Warbler (HOWA; *Setophaga citrina*), Indigo Bunting (INBU; *Passerina cyanea*), Kentucky Warbler (KEWA; *Geothlypis formosa*), while focal species over-wintering in South America were: Red-eyed Vireo (REVI; *Vireo olivaceus*), Gray-cheeked Thrush (GCTH; *Catharus minimus*), Swainson's Thrush (SWTH; *Catharus ustulatus swainsoni*). The over-winter distribution of Swainson's thrush extends across Mexico, Central America, and South America. However, extensive genetic analysis indicates that *C. u. swainsoni*, the subspecies captured at our stopover site, over-winters in South America [35]. Focal species represented a broad range of foraging guilds including: canopy foragers (e.g., REVI), understory foragers (e.g., HOWA, INBU, KEWA), and ground foragers (e.g., WOTH, OVEN, GCTH, SWTH).

**Table 1:** Years during the period of the study (1993 to 1996, 1998 to 2010) categorized as El Niño, La Niña, or non-ENSO years based on average Oceanic Niño Index (ONI) values for the winter time period, December to March.

			Over-winter in Caribbean-Central America					Over-winter in South America		
Year	ONI values	Fall out days	HOWA	INBU	KEWA	OVEN	WOTH	GCTH	REVI	SWTH
El Niño										
1995	1.1	6	38 (15)	75 (21)	30 (10)	31 (17)	40 (18)	13*	152 (62)	18 (8)
1998	2.2	16	142 (48)	212 (142)	99 (52)	94 (61)	117 (97)	103 (65)	182 (128)	120 (69)
2003	1.17	11	172 (59)	184 (73)	112 (35)	73 (22)	95 (66)	50 (8)	46 (14)	72 (22)
2005	0.67	8	74 (7)	115 (53)	46 (9)	63 (32)	145 (99)	51 (15)	224 (105)	171 (75)
2010	1.67	8	80 (34)	661 (374)	72 (43)	51 (30)	188 (181)	8 (5)	105 (49)	198 (101)
La Niña										
1999	−1.3	4	40*	83 (21)	39 (3)	83 (32)	85 (16)	33 (9)	76 (26)	67 (13)
2000	−1.5	16	149 (86)	276 (198)	97 (58)	181 (147)	143 (123)	59 (37)	112 (89)	91 (73)
2001	−0.6	8	108 (38)	197 (79)	102 (56)	64 (34)	81 (41)	15 (5)	103 (54)	24 (4)
2008	−1.37	6	64 (11)	66 (26)	35 (6)	64 (31)	33 (16)	36 (18)	122 (57)	23 (9)
non-ENSO										
1993	0.3	8	30 (5)	134 (30)	40 (12)	40 (11)	62 (93)	9 (4)	155 (71)	49 (15)
1994	0.2	8	7 (4)	95 (50)	28 (15)	31 (14)	13 (9)	25 (9)	101 (65)	37 (19)
1996	−0.4	8	39 (7)	96 (30)	26 (10)	37 (10)	57 (14)	41*	85 (22)	36 (5)
2002	−0.03	6	59 (11)	83 (23)	42 (10)	74 (40)	65 (18)	15 (6)	42 (16)	9*
2004	0.37	7	142 (17)	86 (17)	85 (5)	106 (32)	88 (17)	47 (12)	89 (39)	77 (40)
2006	−0.47	8	28 (6)	133 (82)	11 (3)	51 (30)	24 (9)	44 (17)	46 (20)	48 (17)
2007	0.47	12	159 (72)	71 (21)	47 (17)	48 (22)	64 (25)	22 (11)	92 (45)	48 (18)
2009	−0.4	9	64 (20)	186 (82)	31 (11)	26 (15)	32 (19)	14 (4)	74 (43)	33 (15)

ONI values >0.5 represent El Niño conditions, ONI values <−0.5 represent La Niña conditions, and values >−0.5 and <0.5 are categorized as non-ENSO years.

The number of each species captured in a given year is indicated, followed in parenthesis by the sample size for only fall out days. See text for full bird names.

*Data excluded from analysis for energetic condition because less than 3 birds captured under fall out conditions.

### Spring Migration Variables

We examined three variables, energetic condition, capture rate, and median arrival date, during the migration time period to assess the carry-over effect of ENSO events during the over-winter period on migration. First, given the importance of fat reserves for energy during migration [36] we calculated the energetic condition of focal species at the stopover site by determining the proportion of body mass attributed to fat by subtracting estimates of fat-free mass from body mass measured at capture [37]. Estimates of fat-free mass were calculated utilizing the protocol of Ellegren [38] and Owen and Moore [37]. Larger energetic condition values indicate birds with more fat reserves. During spring migration, a bird's energetic state and/or extrinsic factors such as weather constrain how far inland a migrant will travel after crossing the GOM [31], [32], [39]. Birds with remaining fuel reserves are more likely to migrate further inland to larger more contiguous hardwood forests than birds with reduced energetic states [30]. Therefore, to ensure that our sample was representative of the energetic conditions of all migrants passing through the region, we restricted our analysis to birds captured on “fall out days”, which are days when unfavorable weather conditions (e.g., wind from north, rain) force most birds in both poor and good condition to stop at the stopover site immediately after crossing the GOM [32]. Fall outs typically start in the late morning (range 1000 to 1400) when migrants begin to arrive across the GOM [39], [40], hence we defined a fall out day as having greater than 50 new captures after 1100 hours. Only birds newly captured after 1100 were included in the analysis to avoid inclusion of birds that remained at the site but were not captured on previous banding days [39], [40].

Second, we calculated a rate of capture to assess if potential variation in energetic condition between El Niño and La Niña years resulted in differences in the number of birds utilizing the stopover site. We defined the rate of capture as the number of individuals per focal species captured over an entire migration season for every 100 net hours.

Last, we calculated the median date of arrival for each migration season per focal species to determine if the timing of spring migration varies in relation to El Niño events experienced at over-winter sites. Median date of arrival was utilized because it is unaffected by outliers and therefore would not be biased by extremely early or late migrants within a given year.

### ENSO Classification

We identified years between 1993 and 2010 representing episodes of El Niño or La Niña events during the winter time period utilizing the National Center for Environmental Predictions Oceanic Niño Index (ONI; http://www.cpc.ncep.noaa.gov/products/ analysis_monitoring/ensostuff/ensoyears.shtml), which is a measure of the departure from long-term averages of sea surface temperatures (SST) in the east-central Pacific Ocean, specifically the Niño 3.4 region (5^°^N-5^°^S, 120^°^–170^°^W). The index classifies El Niño and La Niña events based on a threshold of 3 consecutive months above the 0.5°C SST anomaly (El Niño) or below the −0.5°C SST anomaly (La Niña). We examined ONI values between December and March, and identified years meeting the threshold for El Niño or La Niña events during that time period.

### ENSO Impact on Vegetation Vigor

To directly link fluctuations in ENSO weather patterns with changes in habitat conditions experienced by focal species at their over-winter sites in Caribbean-Central and South America, we examined differences in NDVI values between El Niño, La Niña, and non-ENSO years for species-specific over-winter ranges. NDVI values, a measure of vegetation on the land surface assessed from remotely sensed imagery, provide a simple measure of the amount of vegetation related to the level of photosynthetic activity, with higher NDVI values reflecting more rainfall and increased vegetation greenness [41]. We assume that the greenness of the tree canopy as measured by NDVI reflects the overall condition of the habitat experienced by all focal species. We obtained NDVI values from the Global Inventory Modeling Mapping Studies (GIMMS) dataset that spans from 1981 to 2006 [42] and is derived from imagery obtained from the Advanced Very High Resolution Radiometer (AVHRR) sensor onboard the National Oceanic and Atmospheric Agency (NOAA) satellites (15 day composites at 1° resolution). The GIMMS data set is corrected for calibration, view geometry, cloud cover, and other effects not related to vegetation change [43], [44]. We defined over-winter ranges of our focal species in each region using wintering ranges from [45]. Utilizing ArcGIS version 10.0, we obtained NDVI values for each species' over-winter range [45] for each year (1993 to 1996, 1998 to 2006) between the time period of 15 February to 31 March, a critical time period during the nonbreeding season during which migrants prepare for migration [4], [24], [46], [47]. We then calculated a yearly mean NDVI value within the winter range of each focal species from 15 February to 31 March.

### Statistical Analysis

We performed all statistical analyses in R version 3.0.1 (R Development Core Team 2013). Using the R package ‘lme4’ [48] we constructed Linear Mixed Models (LMM) with over-winter NDVI values, energetic condition during migration, capture rates, and timing of migration at the stopover site as response variables. Species (5 species in Central America, 3 species in South America) was included in each model as a random intercept effect to control for distinct differences among species in over-winter NDVI values, energetic condition during migration, capture rates, and timing of migration at the stopover site. First, we tested for a difference in species-specific NDVI values as a function of ENSO event (El Niño, La Niña, or non-ENSO) using LMMs with species as a random factor separately for each over-winter region (South and Caribbean-Central America). Second, for statistical analyses of bird-related response variables, mean values for each species per year were utilized so patterns were not driven solely by large samples in a particular year or species. To determine whether ENSO events influenced energetic condition, capture rate, and median arrival date at a migratory stopover site, we modeled each response variable (energetic condition, capture rate, median arrival date) as a function of ENSO event (El Niño, La Niña, non-ENSO) separately for both Central and South America utilizing a LMM with species as a random factor. We evaluated the significance of models using difflsmeans function in R package lmerTest [49]. The difflsmeans function performs an ANOVA and the approximation for the denominator degrees of freedom of the F statistic is Satterthwaite's, a calculation based on SAS proc mixed theory [49]. For significant models, we calculated parameter estimates and significance of fixed effects with lmerTest [49]. For each model, goodness of fit was confirmed by visual inspection of plots of residuals against predicted values.

## Results

**Figure 1:**
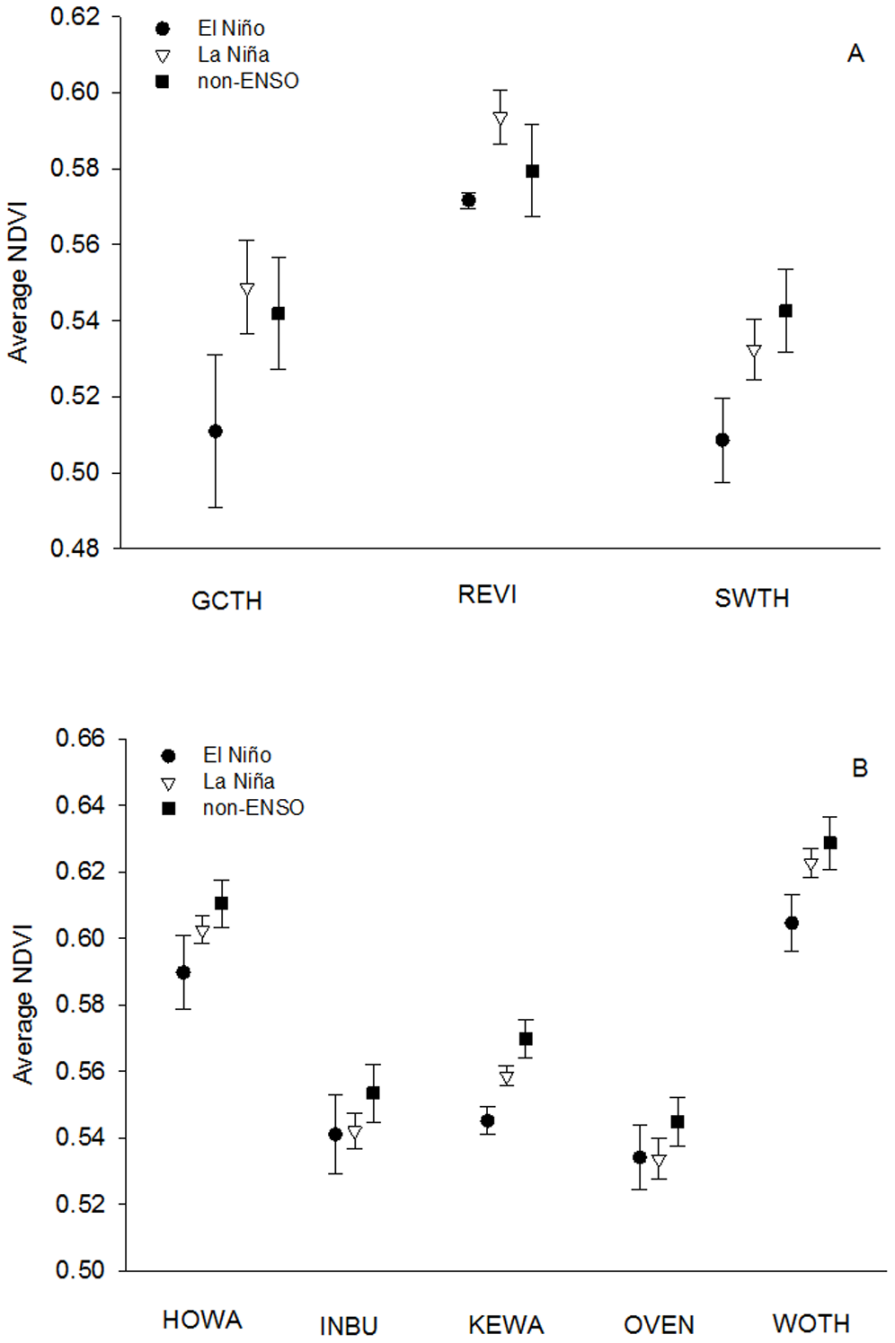
NDVI values for species-specific over-winter ranges and ENSO events. Comparison among El Niño (♦), La Niña (Δ), and non-ENSO (▪) years of average Normalized Difference Vegetation Index (NDVI) values (± SE) for each species' over-winter range between the time period of 15 February to 31 March, a critical time period during the nonbreeding season during which migrants prepare for migration. Average NDVI values between El Niño and La Niña years was significantly different for species over-wintering in (A) South America (El Niño: 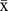 = 0.53±0.01, La Niña: 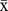 = 0.56±0.01), while average NDVI values during non-ENSO years (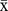 = 0.55±0.008) were consistent with La Niña years. Species over-wintering in (B) Caribbean-Central America did not differ in average NDVI values between El Niño and La Niña years (El Niño: 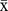 = 0.56±0.008, La Niña: 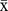 = 0.57±0.009), but both El Niño and La Niña years differed from non-ENSO years (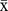 = 0.58±0.007).

Nine years were identified as either El Niño (n = 5) or La Niña events (n = 4) during the time period of the study (1993 to 1996, 1998 to 2010; Table 1). In South America, El Niño events resulted in significantly reduced NDVI values, a measure of vegetation greenness, across each focal species' over-winter range compared to La Niña and Non-ENSO years (Fig. 1a, Table 2; n = 39, F = 3.91, p = 0.03). During non-ENSO years, average NDVI values across species' over-winter ranges in South America did not differ from La Niña years (Fig. 1a, Table 2). As predicted, vegetation greenness did not differ between El Niño and La Niña years in Caribbean-Central America; however, NDVI values were highest during non-ENSO years (Fig. 1b; n = 65, F = 8.09, p = 0.0001).

**Table 2:** Summary of parameter estimates, standard error, t-value, and p-value based on Linear Mixed Models (random effect: species) testing for differences in species-specific NDVI values, a bird's condition at arrival at the stopover site, capture rate (number of birds 100 net hours^-1^), and median arrival date at the stopover site as a function of ENSO category (El Niño, La Niña, and non-ENSO events) for migrants en route from South America.

Model	Paramter Estimate	Standard Error	t	p
NDVI				
ENSO(El Niño)	−0.03	0.01	−2.39	0.02
ENSO(non-ENSO)	−0.003	0.01	−0.34	0.74
Condition				
ENSO(El Niño)	−0.98	0.39	−2.5	0.02
ENSO(non-ENSO)	−0.1	0.36	−0.28	0.78
Capture Rate				
ENSO(El Niño)	0.63	0.29	2.15	0.04
ENSO(non-ENSO)	0.16	0.27	0.59	0.56
Median Arrival				
ENSO(El Niño)	−2.38	2.16	−1.1	0.28
ENSO(non-ENSO)	−4.85	1.11	−2.46	0.02

The value in parentheses following ENSO category indicates the category modeled.

**Figure 2:**
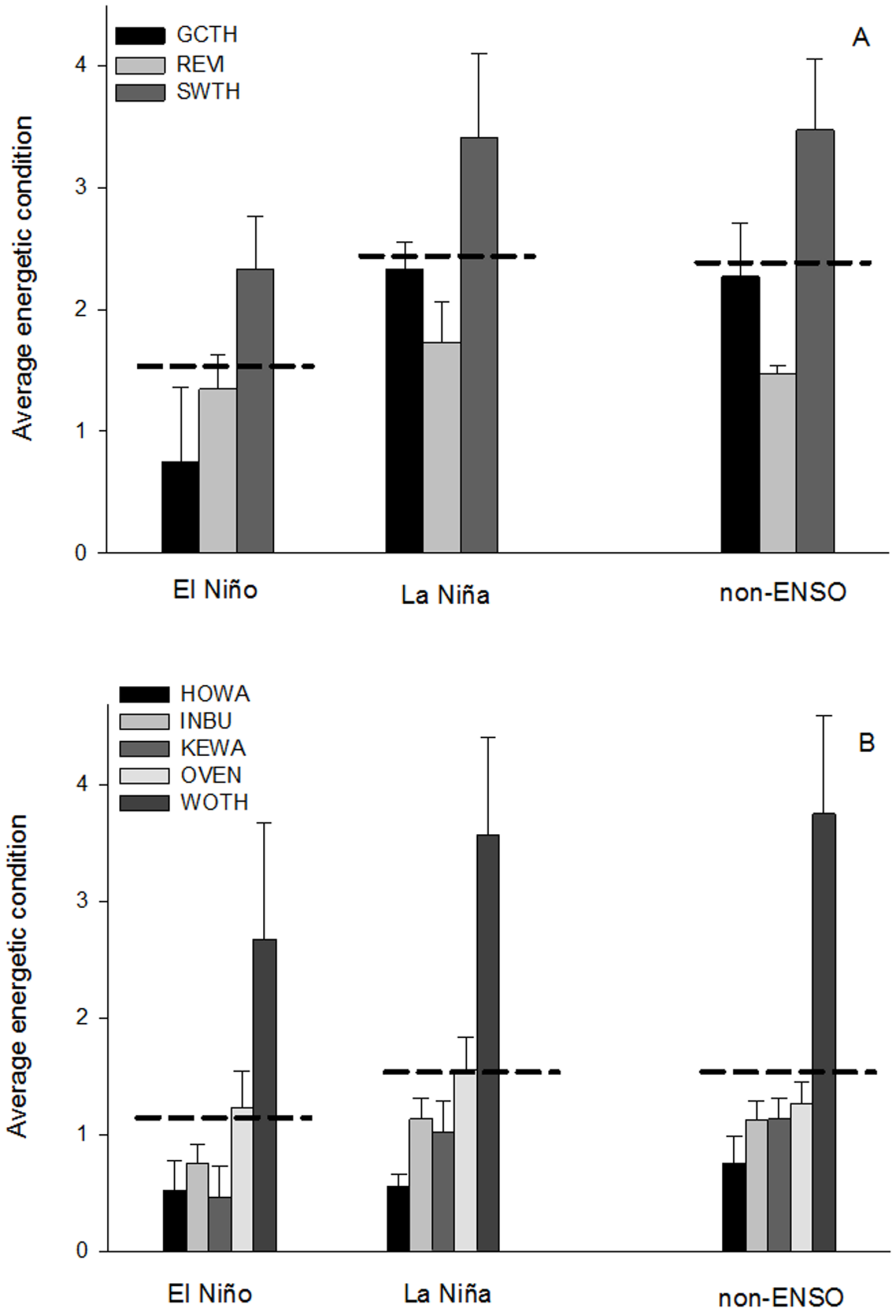
Migratory Energetic condition and ENSO events. Comparison of the average energetic condition of stopover migrant species that over-wintered in (A) South and (B) Caribbean-Central America during El Niño, La Niña, and non-ENSO years. Error bars represent ± SE and dashed lines represent average values for each region (South America: El Niño: 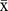 = 1.52±0.29, La Niña: 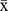 = 2.49±0.32, non-ENSO: 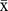 = 2.36±0.29; Caribbean-Central America: El Niño: 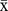 = 1.12±0.26, La Niña: 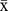 = 1.65±0.32, non-ENSO: 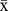 = 1.63±0.24). There were significant differences between El Niño and La Niña years in the average energetic condition for species over-wintering in South America, and average energetic condition during non-ENSO years were consistent with La Niña years. Energetic condition did not differ between El Niño, La Niña, and non-ENSO years for species over-wintering in Caribbean-Central America region.

**Figure 3:**
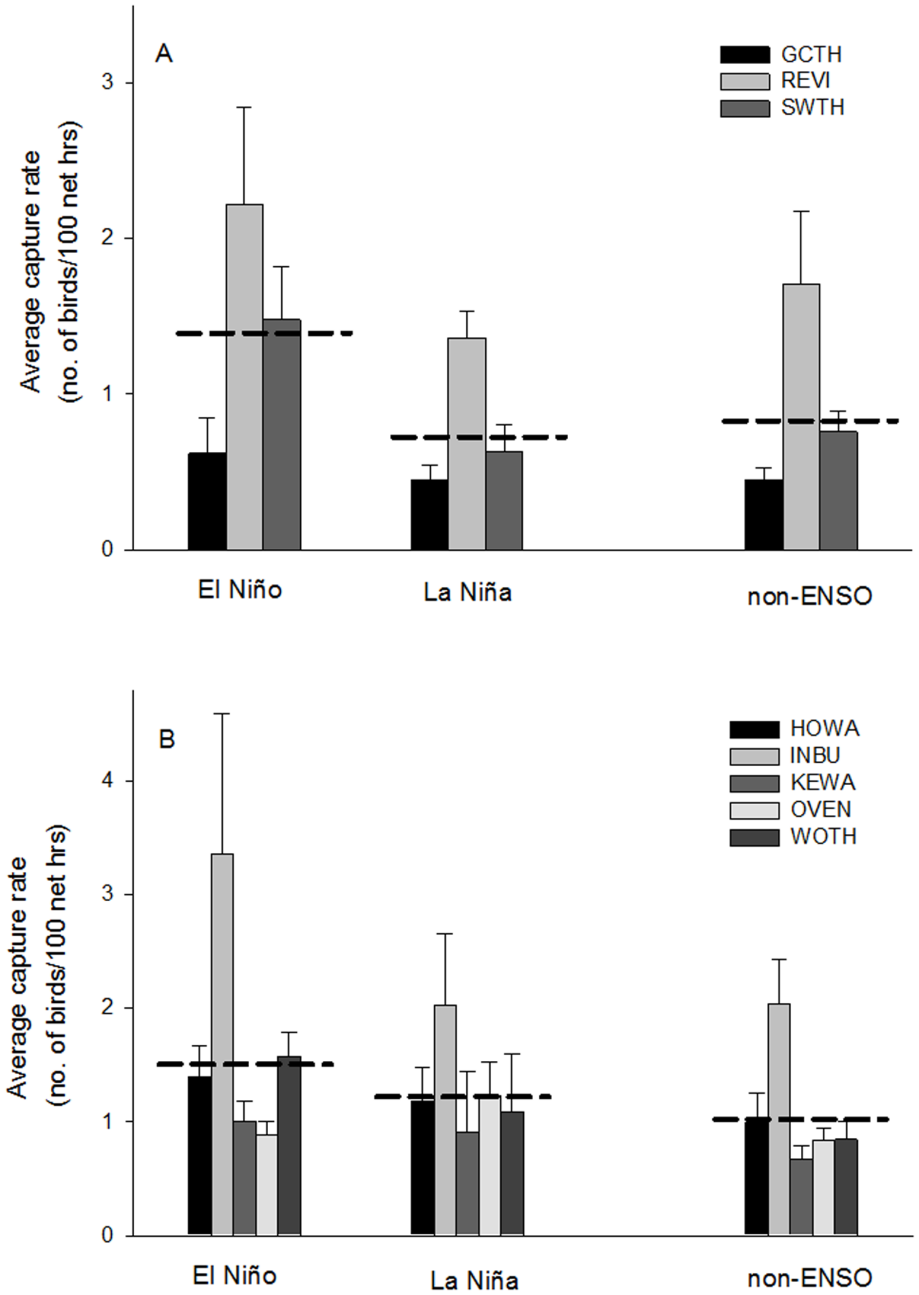
Capture rate and ENSO events. Comparison of average capture rate of stopover migrant species that over-wintered in (A) South and (B) Caribbean-Central America during El Niño, La Niña, and non-ENSO years. Error bars represent ± SE and dashed lines represent average values for each region (South America: El Niño: 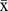 = 1.44±0.29, La Niña: 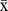 = 0.81±0.14, non-ENSO: 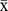 = 0.97±0.19; Caribbean-Central America: El Niño: 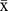 = 1.64±0.30, La Niña: 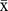 = 1.29±0.17, non-ENSO: 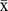 = 1.08±0.13). There were significant differences between El Niño and La Niña years in the average capture rate for species over-wintering in South America, and average capture rate during non-ENSO years were consistent with La Nina years. Capture rates did not differ between El Niño and La Niña years for species over-wintering in Caribbean-Central America region. However, there was a significant difference in capture rates between non-ENSO and El Niño years, primarily driven by an outlier (2010 Indigo Bunting capture rate). The pattern was no longer significant when the outlier was removed (Caribbean-Central America: El Niño: 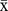 = 1.37±0.13 with outlier removed).

**Figure 4:**
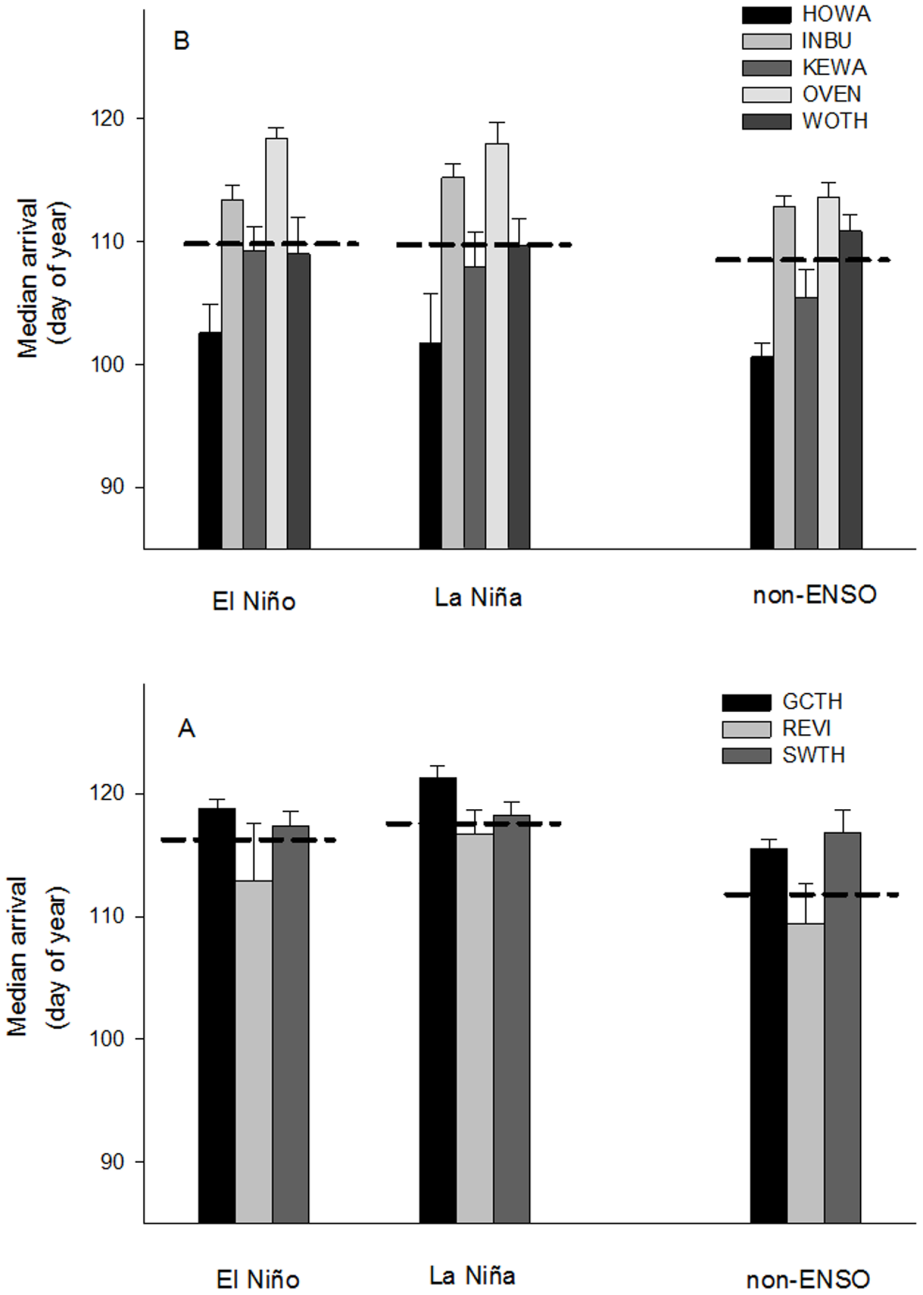
Arrival date and ENSO events. Comparison of median arrival date of stopover migrant species that over-wintered in (A) South and (B) Caribbean-Central America during El Niño, La Niña, and non-ENSO years. Error bars represent ± SE and dashed lines represent average values for each region (South America: El Niño: 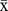 = 116±1.64, La Niña: 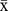 = 118±0.92, non-ENSO: 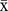 = 113.90±1.41; Caribbean-Central America: El Niño: 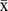 = 110.54±1.34, La Niña: 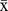 = 110.55±1.66, non-ENSO: 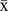 = 108.7±0.99). Range of median arrival dates, April 1  =  Day 91 to May 25  =  Day 125. There was not a significant difference between El Niño and La Niña years in median arrival date for either region.

Consistent with our prediction we found that in El Niño years focal species over-wintering in South America had reduced energetic conditions (Fig. 2a, Table 2; n = 48, F = 4.22, p = 0.02) and consistently higher capture rates at the stopover site (Fig. 3a, Table 2; n = 51, F = 2.71, p = 0.06) compared to La Niña and non-ENSO years. Energetic conditions and rates of capture during non-ENSO years were similar to La Niña years (Fig. 2a & 3a, Table 2). During El Niño years, energetic condition of birds en route from South America was almost half the value of birds captured during La Niña years (El Niño: 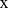 = 1.52±0.29, La Niña: 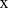 = 2.49±0.32), with twice as many birds utilizing the coastal stopover site (El Niño: 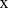 = 1.54±0.29, La Niña: 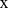 = 0.81±0.14). Contrary to our prediction, reduced energetic condition during El Niño years did not result in a later median arrival date for focal species over-wintering in South America at the stopover site compared to La Niña and non-ENSO years; however, birds arrived earlier during non-ENSO years (Fig. 4a, Table 2; n = 51, F = 3.15, p = 0.05).

In contrast to birds over-wintering in South America, we found that energetic condition (Fig. 2b; n = 83, F = 1.88, p = 0.16) and median arrival date (Fig. 4b; n = 85, F = 1.92, p = 0.15) did not differ between El Niño, La Niña, and non-ENSO years for species over-wintering in Caribbean-Central America. The overall model for capture rate indicated significant differences in capture rates between ENSO events (Fig. 3b; n = 85, F = 3.15, p = 0.05). However, one extreme outlier (INBU 2010 capture rate: 8.17 birds/100 net hours; Table 1) was driving the significant pattern. When we removed the outlier the pattern was no longer significant (n = 84, F = 2.26, p = 0.11).

## Discussion

ENSO events, which cause regional variation in precipitation and temperature, differentially affected migratory birds at over-winter sites in Caribbean-Central and South America with cascading impacts during spring migration. Migratory birds that over-wintered in South America experienced significantly hotter and drier environmental conditions during El Niño years [1], [27], consistent with our NDVI analysis that revealed a reduction in vegetation greenness during El Niño years compared to La Niña and non-ENSO years. In contrast, NDVI values did not differ between El Niño and La Niña years in Caribbean-Central America, which is consistent with only a weak tendency towards drier conditions overall in Caribbean-Central America during El Niño years [1], [27]. Reduced vegetation greenness during El Niño conditions, as reflected by low NDVI values, presumably depresses arthropod abundances [50]–[52] and availability of food resources for migratory birds during the critical pre-migratory period when energy stores are accumulated to fuel migration. Therefore, we expected migratory birds over-wintering in areas where vegetation greenness is influenced by ENSO to depart with lower energetic condition during El Niño years, and our results were consistent with this expectation. We found reduced energetic condition during migration in El Niño years for those migrants over-wintering in South America, and no difference in energetic condition during migration between El Niño, La Niña, and non-ENSO years for migrants over-wintering in Caribbean-Central America.

Despite the complexity of many factors that interact to influence the energetic condition of a migratory bird, we found strong evidence that climatic events during the non-breeding phase of the annual cycle have consequences that carry-over to affect a bird's condition during migration (see also [12], [13]). A bird's energetic state during migration directly impacts how well a migrant negotiates the challenges of migration [7], including aspects of foraging and predator avoidance. Whereas many challenges encountered during migration such as food acquisition and competition for limited resources are similar to challenges faced throughout the annual cycle, the energy demands as well as time and information constraints imposed on birds during the migratory phase amplify these challenges [7], [53]. Therefore, birds departing over-wintering sites with reduced energy stores to meet the considerable physiological challenges encountered en route may experience a higher risk of mortality, especially when crossing an ecological barrier such as the GOM.

Consistent with our prediction, we did not find a clear pattern between ENSO events during the over-winter time period in Caribbean-Central America and a bird's energetic condition during spring migration. However, localized populations within these over-winter regions may have been impacted by El Niño events with carry-over effects during the migratory time period. For example, during El Niño years black-throated blue warblers (*Setophaga caerulescens*) over-wintering in Jamaica had reduced annual survival [3], with the majority of mortality for black-throated blue warblers concentrated during the migratory time period [14]. While the overall impact of ENSO events in the Caribbean and Central American is weak compared to other regions [27], the extent to which areas within the Caribbean and Central America are affected by ENSO conditions varies [29]. Understanding the impacts of ENSO conditions in the Caribbean and Central America is further complicated by the topography of the region, which results in contrasting climatic conditions along the Caribbean (increased rainfall) and Pacific (decreased rainfall) coasts during El Niño events [28], [29]. The impact of El Niño events on some populations over-wintering in the Caribbean-Central America is potentially diffused at our study site by the diversity of breeding populations from a wide range of over-winter regions utilizing coastal habitats on the northern GOM.

Energetically constrained migrants are often forced to make landfall in the first available habitat encountered after crossing an ecological barrier, regardless of suitability (e.g., [54], [55]). During El Niño years, birds en route from South America were twice as likely to use our stopover site located immediately along the Gulf coast after crossing the GOM. Within the heavily developed Gulf coast region a mosaic of small isolated patches of habitat provide migratory birds with a refuge to rest after crossing the GOM. However, food resources to replenish fat and muscle reserves within these small patches are lower than in larger more contiguous hardwood forests further inland [30]. Moreover, as birds concentrate within these small-forest patches, competition for already limited resources increases and fuel deposition rates decline [56], [57]. The consequences of reduced energetic condition during stopover that result from adverse climatic events experienced during the over-winter period during El Niño years may be compounded by landfall in less suitable habitat, and impose additional costs during the migratory time period with cascading impacts on survival and future reproductive performance.

The median arrival date was remarkably consistent during the 17-year study (see also [58]), despite climatic variability during the over-winter phase of the annual cycle, suggesting that there is a strong drive for birds to maintain their time program during migration [22], [59], [60], likely in response to the enhanced reproductive success of early arriving individuals [21], [25], [61], [62]. This consistency is contrary to our prediction that the cascading impact of reduced energy stores for birds over-wintering in South America during El Niño years would result in later arrival at stopover sites along the Gulf coast. Whereas transitions between different phases of the annual cycle may be controlled by an endogenous time program synchronized with seasonal changes in photoperiod [63], there is evidence that spring migration is phenotypically plastic among some intercontinental migratory species, and climatic conditions such as reduced precipitation at nonbreeding sites may delay the timing of spring departure [46], [62], [64]. If a bird is to maintain its endogenous time program we might expect initiation of migration to vary little from year to year, even when climatic events depress arthropod abundances, and consequently affect the margin of safety in energy reserves for departing migrants. That said, if migrants are not able to compensate for the reduced energy reserves during the migratory period, they are likely to arrive at their breeding grounds without surplus fat stores, and may suffer reproductive consequences [65]. Alternatively, El Niño events may delay spring departure from over-wintering areas as birds forage longer to gain sufficient energy stores, forcing birds to increase their speed of migration to stay on schedule with their time program. While few studies have been able to quantify the rate of migration for small landbirds, given the challenges of following individuals across large spatial scales (but see [66], [67]), evidence suggests that birds can adjust the rate of migration in response to ecological conditions during migration [26], [68], [69]. However, even with delayed departure from the nonbreeding grounds, birds may still have reduced energy reserves if arthropod abundances are sufficiently depressed.

If we are to understand how climatic events impact migratory populations, we must integrate the cumulative effects of events that occur during different phases of the annual cycle. While studies have found associations between ENSO events on the breeding and nonbreeding grounds with annual survival, productivity, and recruitment [3], [9], [10], rarely have studies considered the influence of the migratory period on the overall annual cycle. Yet, the negative impacts of climatic events during the stationary, nonbreeding phase of the annual cycle may not be manifested until migrants are faced with the physiological demands of migration. Climatic models predict that the frequency and intensity of ENSO events will increase in the coming decades [17], [18], [19]. Increased El Niño events, coupled with the loss of stopover habitat, especially within coastal regions [31], may intensify the challenges of migration, increasing the risk of mortality during migration. The differential impact of El Niño events on the migratory condition of birds over-wintering in Caribbean-Central and South America, suggests that more frequent El Niño events may disproportionately influence long-distance migrants over-wintering in South America. We may expect that La Niña years, which have the opposite climatic effects (e.g., wet and cool) of El Niño conditions in South America [1], [27], might provide a positive response to birds to offset the negative effects of El Niño years. This prediction is consistent with studies that found higher reproductive success for breeding birds in North America during La Niña years, presumably due to increased food resources [3], [10]. Yet, we found that the energetic condition of birds over-wintering in South America during La Niña years largely overlapped with non-ENSO or ‘normal years’, as did NDVI values, suggesting that increases in rain above a certain level may not necessarily increase vegetation greenness and food resources for bird species over-wintering in the tropical forests of South America. Therefore, increased frequency of ENSO events are likely to have mostly negative impacts on the population dynamics of bird species over-wintering in South America.

Our understanding of the migratory time period has been limited by the difficulty of determining a migrant's geographic linkage to over-wintering and breeding areas and the conditions a migrant experiences prior to arriving at a stopover site. Yet, the phases of a migrant's annual cycle are inescapably linked. There is increasing evidence that late winter climate influences the time of arrival on breeding grounds and subsequent reproductive success for long-distance migrants [4], [24], [47], [70], but little is know about how climate during the over-winter period may influence migrants during migration, the phase of the annual cycle most often thought to be limiting migratory bird populations [14]–[16]. We provide strong evidence that not only are migratory birds during spring migration influenced by events occurring during the previous phase of their annual cycle, but also where they over-winter determines how vulnerable they are to global climatic cycles. We directly associate climatic variability experienced at over-winter areas with factors important to the success of migration, namely timing and condition, providing important insight into how global climatic cycles such as ENSO influence migratory birds throughout the annual cycle. Predicting how migratory species will adapt to a changing climate, including increased frequency and intensity of ENSO events [17], [18], requires understanding how climate effects each phase of the annual cycle and how those effects carry-over to subsequent phases.
